# Comparative assessment of myocardial function between late premature newborns and term neonates using the 2D speckle tracking method

**DOI:** 10.3389/fped.2024.1302383

**Published:** 2024-03-14

**Authors:** Daniela Toma, Amalia Făgărășan, Andreea Cerghit-Paler, Rodica Togănel, Manuela Cucerea, Maria Oana Săsăran, Liliana Gozar

**Affiliations:** ^1^ Department of Pediatrics III, Faculty of Medicine, George Emil Palade University of Medicine, Pharmacy, Science, and Technology of Târgu Mureș, Târgu Mureș, Romania; ^2^ Department of Pediatric Cardiology, Emergency Institute for Cardiovascular Diseases and Transplantation, Târgu Mureș, Romania; ^3^ Department of Pediatrics IV, Faculty of Medicine, George Emil Palade University of Medicine, Pharmacy, Science, and Technology of Târgu Mureș, Târgu Mureș, Romania; ^4^ Department of Neonatology, County Emergency Hospital Targu Mures, Târgu Mureș, Romania

**Keywords:** premature newborn, speckle tracking, myocardial function, strain analysis, longitudinal deformation

## Abstract

**Introduction:**

Assessment of myocardial function through speckle tracking echocardiography (STE) can bring benefits to conventional echocardiography in premature newborns, a particular vulnerable group in terms of adaptation to extra-uterine life. Furthermore, it represents a non-invasive imagistic method which can guide therapeutic approach in the hemodynamically unstable newborn. This study aims to highlight the particularities of myocardial function in late premature newborns, by conducting a comparison with a group of healthy neonates, by using STE.

**Methods:**

Conducted over a timespan of two years, this prospective study enrolled 64 term neonates and 21 premature newborns, with gestational ages ranging between 28 and 36 weeks, who prior to discharge underwent a cardiac ultrasound, involving two-dimensional image acquisitions of the apical four-chamber view of both ventricles. Afterwards, the images were offline analyzed, by using the autostrain function.

**Results:**

After segmental strain analysis, no significant discrepancies between the two groups in terms of interventricular values were found. However, left ventricle and right ventricle strain measurements differed significantly (*p* < 0.01), for each of the analyzed segments (basal, medial or apical). Moreover, a linear increase in interventricular (IV) basal strain with corrected gestational age progression was noted (*p* = 0.04). Peak global longitudinal strain (pGLS) and EF were similar between the two study groups. Premature newborns presented significantly more negative mean values of right ventricular free wall longitudinal strain (RVFWSL), (−24.19 ± 4.95 vs. −18.05 ± 5.88, *p* < 0.01) and of right ventricle global four chamber longitudinal strain (RV4CSL), (−19.71 ± 3.62 vs. −15.46 ± 5.59, *p* < 0.01), when compared to term neonates.

**Conclusions:**

The 2D STE is a reliable method for cardiac assessment of late preterm newborns. The evaluation of two-dimensional global longitudinal LV and RV strains might represent a useful tool in clinical practice. A better response of the right ventricle to the longitudinal deformation within premature neonates was noted. Thus, this study facilitates the identification of accurate reference values for this particular population segment, which will enable the evaluation of ventricular function in premature newborns with concurring disorders. Future longitudinal studies, assessing the fetal heart, could provide more insight into the development of myocardial function.

## 1 Introduction

The hemodynamic evaluation of newborns during the complex, transitional phase to neonatal life still remains a subject of great interest, due to the paucity of available literature data. Premature newborns are constantly undergoing hemodynamic challenges during the postnatal period (1). The adaptation process in premature newborns is influenced by several factors, such as the timing of umbilical cord clamping, medication, maternal co-existing conditions and organ immaturity. Moreover, myocardial function is altered by hypoxia, sepsis and intrauterine growth retardation (2). Cardiac ultrasonography remains one of the important non-invasive tools which assesses myocardial function and can simultaneously offer data for the proper management of the hemodynamically instable newborn, as well as information related to the monitoring of their evolution and prognosis. Advanced cardiac ultrasound techniques such as the analysis of myocardial deformation through speckle tracking (STE) have been proven to be superior to conventional measurements of myocardial function, in terms of identifying myocardial disfunction, characterizing myocardial phenotype and guiding treatment (3, 4). Reference intervals for myocardial deformation are mostly found in term newborns and extremely premature newborns, with scarce data available in mid to late premature newborns. This particular age group is vulnerable through respiratory morbidity and pulmonary hypertension risk (5).

Thus, our study aims to describe characteristics of myocardial function in late premature newborns, through a comparison conducted with a group of healthy term neonates, with the help of STE.

## 2 Material and methods

### 2.1 Study population

The current prospective study was conducted within a timespan of two years (January 2020-April 2022), enrolling patients admitted to the Pediatric Cardiology Clinic III, an integrated part of the Emergency Institute for Cardiovascular Diseases and Transplantation Târgu Mureș. The inclusion criteria consisted of hemodinamically stable premature neonates at the moment of examination, referred for cardiological consult due to an audible systolic murmur, with gestional ages ranging between 28 and 36 weeks (group 2), whereas the control group was composed of healthy, appropriate for gestational age (AGA) neonates, born on term (group 1). Neonates with genetic syndromes, congenital cardiac or non-cardiac malformations were excluded, with the exception of those who presented insignificantly hemodynamic anomalies, such as patent foramen ovale (PFO), patent ductus arteriosus (PDA) or atrial septal defect (ASD). Premature neonates, with serious complications which could have impacted cardio-pulmonary function (respiratory distress syndrome, severe/prolonged jaundice, sepsis, moderate/ hemodynamically significant PDA, necrotizing enterocolitis) were left out of the study. For both study groups, maternal gestational diabetes constituted another exclusion criterium, whereas for the control group, newborns with signs of infection, renal injury or other comorbidities were ruled out. Four premature neonates in whom STE evaluation could not be performed based on the ultrasound images were excluded.

### 2.2 Cardiac ultrasound image acquisition and STE analysis

Each echocardiography was performed with the help of a Philips Epiq 7 ultrasound machine, prior to hospital discharge. The timing of echocardiography examinations corresponded to an age of 2–4 days of life for group 1 and to an age ranging between 5 and 54 days for the premature study group. Bidimensional, optimal quality image acquisitions, typical for the apical 4 chamber view (2D STE), within a frame rate of over 70 Hz, were deposed in a DICOM format and afterwards offline analyzed, using the right and left ventricle autostrain functions of the Philips QLAB 15 software. In order to perform an analysis of the entire cardiac cycle, its M mode depiction coincided with the closing and opening of the mitral valve, which is automatically generated by the software. For automatic drawing of the endocardium, three points were marked, at the base of the septum, lateral and apical. Afterwards, the drawing was verified and manually corrected.

Therefore, cardiac ultrasound images were analyzed by a sole investigator, the ejection fraction (EF) was measured through left ventricle M mode, together with the global longitudinal strain (pGLS) and the biventricular segmental strain, as the interventricular septum and the walls were divided into three segments (left ventricle-LV Basal, LV Medial, LV Apical, interventricular-IV basal, IV Medial, IV Apical, right ventricle- RV Basal, RV Medial, RV Apical, right ventricular free wall longitudinal strain- RVFWLS, right ventricle global four chamber longitudinal strain- RV4CLS). The aforementioned parameters were compared between the two study groups.

### 2.3 Statistical analysis

Parameters such as mean, frequency and standard deviation were expressed as part of descriptive statistics. In order to establish type of distribution of the analyzed data, Kolmogorov-Smirnov test was applied. The results of this normality test drove the appropriate approach for mean comparison of quantitative variables, which involved the use of the Mann–Whitney test (for variables non-compliant with a Gaussian distribution) or of the unpaired t-Student test (for variables with a Gaussian distribution). In case of categorical variables, chi-square test was applied and odds ratios (ORs) were obtained. Simple linear regression tried to establish a correlation between each strain parameter obtained and corrected gestational age progression for the premature neonatal group. In case of each analyzed variable, a confidence interval (CI) of 95% was used as reference, and therefore *p*-values of under 0.05 were considered statistically significant. The entire statistical analysis was conducted with the help of the GraphPad Prism T 9.0 software.

### 2.4 Ethics

The research was conducted in accordance with the principles stated in the declaration of Helsinki. Prior to inclusion in the study, a signed, informed consent form was obtained from at least one of the legal tutors of each child. The research protocol was approved by the Ethics Committee of Emergency Institute for Cardiovascular Diseases and Transplantation Târgu Mureș and by the one of the George Emil Palade University of Medicine, Pharmacy, Science, and Technology of Târgu Mureș (approval no. 1276/25.02.2021).

## 3 Results

After exclusion of 5 patients in whom the parents/legal tutors refused to sign the informed consent form, the study included 64 term neonates (group 1) and 21 pre-term neonates (group 2). Descriptive data and comparation of the baseline characteristics of the two groups are represented in Table 1. There were no significant differences between the two groups in terms of gender distribution (*p* = 0.41), but a higher prevalence of caesarian section type of delivery was found in the premature group (*p* < 0.01, OR = 9.93). Mean birth weight and length were expectably lower in the premature neonate group (3,450 ± 283 gr. vs. 1,815 ± 415.3 gr., *p* < 0.01 and 54.81 ± 2.26 cm vs. 43.52 ± 4.87 cm, *p* < 0.01). Gestational age at the moment of birth was 32.76 ± 2.80 weeks in group 2, as opposed to 38.97 ± 0.79 weeks in group 1 (*p* < 0.01). The premature neonates were examined at a mean corrected gestational age of 35.57 ± 1.43 weeks, significantly later after birth than mature newborns (*p* < 0.01, Table 1). An important percentage of these premature neonates (42.85%) came from twin pregnancies.

**Table 1:** Baseline characteristics of the two neonatal study groups.

Neonatal characteristics		Group 1^a^ (*n* = 64)	Group 2^a^ (*n* = 21)	*P*-value
Gender (%)	Female	37.5	47.61	0.41, OR=0.66 (95% CI: 0.25–1.67)
	Male	62.5	52.38	
Type of delivery (%)	Vaginal delivery	85.93	38.09	<0.01, OR=9.93 (95% CI: 3.35–29.36)
	Caesarian section	14.06	61.90	
Birth weight (gr.)^b^		3,450 ± 283	1,815 ± 415.3	<0.01
Length (cm)		54.81 ± 2.26	43.52 ± 4.87	<0.01
Gestational age at birth (weeks)		38.97 ± 0.79	32.76 ± 2.80	<0.01
APGAR score at 1 min.^b^		9.31 ± 0.66	7.23 ± 2.32	<0.01
Age at the time of echocardiography (days)		2.89 ± 0.79	19.62 ± 12.91	<0.01

CI, confidence interval; cm, centimeter; gr, grams; OR, odds ratio.

^a^ data has been expressed as mean ± SD (standard deviation) for quantitative variables and % for categorical variables.

^b^ Unpaired *t*-test was applied (variables complying to a Gaussian distribution).

**Figure 1:**
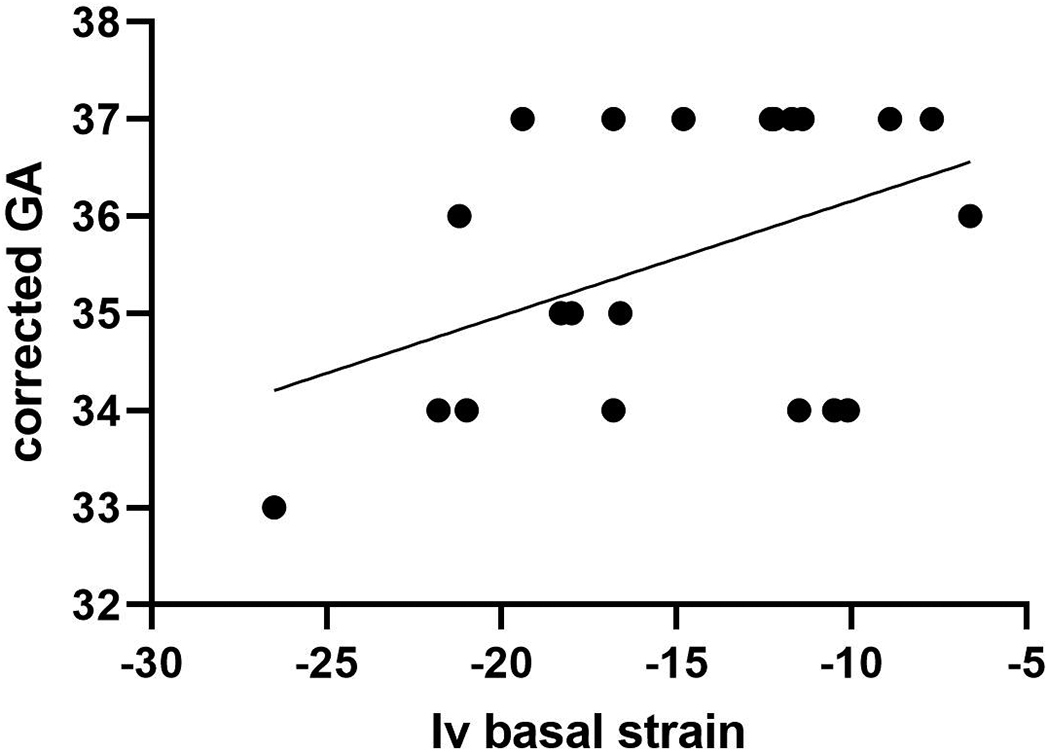
Relationship between Iv basal strain measurements and gestational age progression among group 2.

Segmental strain analysis revealed no significant differences between the two groups in terms of interventricular values. Significant discrepancies between the two groups in left ventricle and right ventricle strain measurements were found (*p* < 0.01), for each of the analyzed segments (basal, medial or apical). These results have been depicted in Table 2. Linear regression analysis was performed in order to establish a possible relationship between segmental strain measurements and gestational age within group 2. A linear increase in interventricular (IV) basal strain with corrected gestational age progression was observed, as pictured in Figure 1 (*p* = 0.04). The other segmental strain parameters did not present significant variations in relation to corrected gestational age.

**Table 2:** Longitudinal segmental strain value comparison between the two study groups.

Segmental strain ratio		Group 1 (mean % ± SD)	Group 2 (mean % ± SD)	*P*-value
IV	Basal	−14.17 ± 5.72	−14.96 ± 5.28	0.48
	Medial	−18.89 ± 3.57	−19.88 ± 5.82	0.69
	Apical^a^	−26.40 ± 6.54	−26.94 ± 5.16	0.72
LV	Basal^a^	−27.94 ± 10.57	−21.32 ± 6.26	<0.01
	Medial^a^	−11.83 ± 5.27	−16.70 ± 4.81	<0.01
	Apical^a^	−18.88 ± 7.03	−23.13 ± 5.89	0.01
RV	Basal	−19.65 ± 7.19	−26.13 ± 4.91	<0.01
	Medial	−16.67 ± 5.53	−22.04 ± 6.03	<0.01
	Apical	−16.46 ± 6.60	−21.91 ± 5.44	<0.01

IV, interventricular; LV, left ventricle; RV, right ventricle; SD, standard deviation.

^a^ Unpaired *t*-test was applied (variables complying to a Gaussian distribution).

Peak global longitudinal strain (pGLS) and EF presented similarities between the two study groups (*p* = 0.32 and *p* = 0.87, respectively). Significantly lower mean % values of RVFWSL were found within preterm neonates, as opposed to mature ones (−24.19 ± 4.95 vs. −18.05 ± 5.88, *p* < 0.01). Furthermore, important decrease in mean % values of RV4CSL were found in group 2 when compared with group 1 (−19.71 ± 3.62 vs. −15.46 ± 5.59, *p* < 0.01). These numeric data have been depicted in Table 3. As in the case of segmental strain, linear regression was applied to establish a relationship between each of the aforementioned longitudinal strain parameters and corrected gestational age, but this analysis retrieved no significant statistical findings.

**Table 3:** Longitudinal left and right ventricular strain parameter comparison between the two study groups.

	Group 1 (mean % ± SD)	Group 2 (mean % ± SD)	*P*-value
LV pGLS	−19.19 ± 3.24	−20.66 ± 3.73	0.32
M mode EF^a^	66.95 ± 4.83	66.76 ± 4.25	0.87
RVFWLS	−18.05 ± 5.88	−24.19 ± 4.95	<0.01
RV4CSL	−15.46 ± 5.59	−19.71 ± 3.62	<0.01

EF, ejection fraction; pGLS, peak global longitudinal strain; LV, left ventricle; RV4CSL, right ventricle global four chamber longitudinal strain; RVFWLS, right ventricular free wall longitudinal strain; SD, standard deviation.

^a^ Unpaired t test was applied (variables complying to a Gaussian distribution).

## 4 Discussions

This study aimed to analyze the effect of premature birth upon myocardial function. In the first few days of life, ventricular adaptation normally occurs, which causes major shifts in tissue velocities (6). Studies performed on animals revealed that the exposure of the premature heart to high flow resistances and to a hypoxemic environment causes significant myocardial hypertrophy and increase in interstitial collagen fibres, while the number of myocytes stays the same (7). Premature neonates present an augmentation of the left ventricular mass and the left ventricular mass/body surface area ratio in comparison with term homologues. Consequently, a volume reduction of the left ventricle takes place, which, dependently upon the severity of prematurity, is associated with a reduction in systolic, diastolic and rotation function, according to Lewandovski et al. (8). The same author proved through another study that severe prematurity is linked to structural and functional alterations of the right ventricle. The EF and the volume of the right ventricle, determined through magnetic resonance imaging (MRI), are lower in adults who were born prematurely, but at the same time their right ventricular mass is larger in size (9). However, one study that comparatively assessed preterm and term neonates, who underwent serial 2-dimensional echocardiography, as well as Doppler trans-mitral flow velocity assessments, revealed that the strongest discrepancies for premature newborns were in those parameters of diastolic function, whereas systolic function measurements maintained within normal ranges. Therefore, the authors concluded that, in premature newborns, diastolic patterns reflect a transition between fetal life and term neonates (10).

Premature newborns also present several specific pathologies, such as hemodynamically significant PDA, pulmonary conditions which cause hypoxemia, including bronchopulmonary dysplasia. These conditions increase in frequency among the extremely premature infants (11, 12). As a result, ventricular filling changes and an increase in afterload occurs, especially for the right ventricle (12). As we have seeked to establish reference values which are not influenced by prematurity- associated pathology, we excluded from our study premature newborns with sepsis, pulmonary hypertension, bronchopulmonary dysplasia, persistent ductus arteriosus, or other cardiac malformations with hemodynamic impact. Concurring pathologies can also influence longitudinal strain values in term newborns. One study showed that newborns with hyperbilirubinemia present significantly lower values of stratified strain parameters of the ventricular wall when compared to healthy, age-matched controls. Still, the two groups were no different in terms of GLS values and its derived parameters (13). Within our study, newborns with prolonged, severe jaundice were left out of the study. Although the age discrepancy between the two study groups at the time of the cardiac ultrasound examination was significant, Klitsie et al. observed that age does not influence left ventricular longitudinal speckle tracking values within their study carried out on term newborns. No significant differences were found in the comparative assessment of the left ventricle performance at the age of 3 days, 3 weeks and 6–7 weeks of life (14).

More insight into premature myocardial function was gained with the addition of speckle-tracking to cardiac ultrasound assessment, which has been regarded as superior to conventional and tissue-Doppler derived echocardiography in healthy newborns and infants (15). The possibility to expand its application in premature neonates derives from great feasibility of tissue Doppler-derived deformation parameters, which can be obtained with adequate imaging quality (16). A greater reproducibility was however reported in relation to basal longitudinal strain and strain ratio measurements of the interventricular septum and right ventricle, whereas a wider range of values was found for the same measurements performed for the left ventricle (16, 17). Some of the limitations of speckle-tracking include the angle effect, which is more prevalent in apical myocardial segment analysis and is closely related to tissue Doppler imaging (18, 19). Elkiran et al. also emphasized the role of aberrant bands, which greatly influence left ventricular form and subsequent deformation-derived parameters (15). Frame rate of image acquisition can also influence ventricular longitudinal deformation imaging in premature infants, which could impact intra-observer and inter-observer reliability (20).Within our study, reproducibility of strain-derived parameters in premature newborns could not be assessed, as image acquisition was performed by a unique examinator.

In normal healthy children (with ages ranging from 0 to 19 years), mean LV pGLS values of −20.5 (95% CI, −20.0 to −21.0) were globally obtained, according to a meta-analysis performed by Jashari et al., which included multiple paediatric age groups (21). However, discrepancies can be found between various small scale neonatal studies, which have reported mean LV pGLS values of −18.8 (95% CI, −20.1 to −17.5), −19.7 (95% CI, −20.8 to −18.5) and −24.5 (95% CI, −25.4 to −23.5). The small population samples included cannot be overseen, as these could have greatly influenced the reference intervals of the studied parameters (14, 22, 23). The pGLS mean obtained within our study is though similar to one of our previous works, which included a compellingly higher study population, of 103 term neonates (24). Still, previous studies mostly enrolled term neonates and did not seek to provide referral ranges of speckle tracking derived parameters for premature newborns.

Within this study, the functional analysis of the left ventricle revealed no alterations, as the EF remained within normal ranges for both study groups. The values of the longitudinal deformity parameter, pGLS, were very similar between the two study groups, although the segmental strain values were more negativein the premature group than in mature newborns, with one exception (the basal segment of the left ventricle). However, in premature newborns, the values for both parameters which assessed the longitudinal deformation of the right ventricle, namely RVFWLS and RV4CSL, were significantly more negative when compared to mature newborns (*p* < 0.01). These findings suggest that the right ventricle of the premature responds better to longitudinal deformation, which is indicative of a better compliance. It is well known that, during the last trimester of pregnancy, a ventricular disproportion takes place, with the predominance of the right ventricle. This phenomenon occurs due to the hemodynamic changes which are typical for the last period of pregnancy: narrowing of the arterial duct, increase in pulmonary flow concomitantly with increase in pulmonary venous return, reduction of the right-left shunt through the foramen ovale, increase in right ventricle afterload (25). Premature birth eliminates this physiological heart overload or diminishes its duration, which could explain why longitudinal strain values are higher in the premature newborns, when compared with term neonates. Therefore, our study provided a unique insight into the understanding of the peculiarities of ventricular compliance of the premature newborns, as opposed to mature newborns. It is though worth mentioning that term newborns included in the study were evaluated in the first 2–4 days of life, which represented the beginning of the transition period from fetal circulation towards postnatal circulatory patterns, and this aspect has been reflected by the reported results.

Within the present research, strain analysis of the interventricular segments did not result in any significant differences between the two study groups, but the apex to base gradient is quite obvious, and this key element had already been highlighted by individual studies and meta-analyses conducted on both pediatric and adult populations (26). Moreover, within our study, in accordance with previously published data (23), segmental strain values of the right ventricle showed a basal-apex gradient decrease, within both study groups.

Strain ratio measurements have been uniformly lower in preterm neonates than in term counterparts, according to a review of literature data (17). These results mostly comply to our findings of right and left ventricular segmental strain measurements, with the exception of the left ventricular basal strain, which actually showed significantly higher values in the preterm neonatal group. Moreover, within our study we found no significant shifts in interventricular strain measurements between the two target groups. We further proceeded to seek possible correlations between strain measurements and corrected gestational age at the time of the examination within the premature population. Interventricular basal strain was the only one which showed an ascending trend with corrected gestational age increase. Previous studies support a lack of significant maturational changes in the first weeks of live for basal deformation parameters (27, 28). Still, variations of segmental strain ratio in relation to gestational age have not yet been reported.

This study enriches literature data through the identification of referral values of longitudinal, global and segmental strain for both ventricles in premature newborns, with gestational ages ranging between 28 and 36 weeks. The establishment of referral values for speckle tracking measurements can aid in a better understanding of the transitional physiology of myocardial function in premature newborns (1) and could aid in the premature identification of subclinical cardiac dysfunction (3). Moreover, the discovery of a better response of the right ventricle to the longitudinal deformation in premature neonates, as opposed to term newborns, enables the knowledge of accurate reference values for this particular population segment, which will create the premises for the evaluation of ventricular function in premature newborns with concurring disorders. Still, the limitations of the current study cannot be foreseen. The main limitation of the current study is the inclusion of a relatively small number of premature neonates. Another limitation of the current study is the age discrepancy at the moment of echocardiographic evaluation between the two groups. Therefore, our results cannot mirror the transition process which could influence the speckle tracking values, and which should be longitudinally evaluated. Therefore, the current study enforces future research which will evaluate myocardial function. Moreover, longitudinal studies, initiated during intrauterine life could provide more insight into the development of myocardial function.

## 5 Conclusions

The 2D speckle tracking method can confidently assess cardiac function of late preterm newborns. Two-dimensional global longitudinal LV and RV strains may represent a possible alternative for the cardiac assessment of preterm newborns in the clinical practice. For assessment of cardiac function, both parameters can be used (LVpGLS to assess LV function, respectively the RVFWSL and RV4CSL for the RV function). The reference values obtained for the LV myocardial function, LVpGLS are between [−20.66 ± 3.73], and those for the RV RVFWSL are [−24.19 ± 4.95], respectively RV4CSL [−19.71 ± 3.62].

## Data Availability

The raw data supporting the conclusions of this article will be made available by the authors, without undue reservation.
